# Notes and News

**Published:** 1894-01-20

**Authors:** 


### Notes and News.

Collected and Communicated.

A paper read by Dr. J. H. Mason before the American Climatological Association made a most valuable suggestion. He refers to the difficulty of finding accommodation in the country, seashore, and elsewhere for patients suffering with whooping-cough, and his suggestion is that a central bureau should be formed in every city, at which could be registered the addresses of those willing to receive such patients. He regards the removal of children suffering from pertussis to a pure atmosphere as an important factor in recovery.

We have received an appeal, signed by the Lord Chancellor, the Duke of Westminster, Sir J. Crichton-Browne, &c., asking for public support for the National Society for the Employment of Epileptics. Thanks to the generosity of Mr. Passmore Edwards, the society has been enabled to purchase a farm for the proposed industrial colony, comprising 135 acres, situated on a high ground at Chalfont St. Peters, Bucks. Funds are in hand to provide for about a score of patients; but, as every medical man knows well, persons in a position to take advantage of such opportunities as the colony offers are to be numbered by hundreds.

Eggs are unquestionably excellent and useful items of food, and fresh English eggs are, of course, better than the imported article. We are not, however, quite sure if the correspondent of the papers who is now advocating town kept hens should be regarded in the light of a philanthropist. His recommendation applies to those who have sufficient air and space to keep fowls. Experience shows that views on the subject of suitable areas certainly differ. A near neighbour of ours regards a small front area as a sufficient run, with night quarters in the coal cellar. When night quarters are inhabited until reasonable hours in the morning, the objection to this feathered population is not apparent more than a few yards off; but when the morning exercise begins early, the tired worker roused from well earned sleep, is far from charitably disposed towards home produce in the shape of eggs, and is inclined to think that the other side of the Channel is better as a fowl run than the streets of the metropolis.

The last district to plead against the erection of a fever hospital in its vicinity is Ealing. A site is required for a hospital for the joint parishes of Acton, Chiswick, and Hanwell. Ealing considers that it is already at a disadvantage by the possession of two cemeteries in its neighbourhood.

The hospital at Torbay which has been undergoing repairs is now reopened, and may be practically regarded as a new hospital. Accommodation for sixty patients and twelve nurses is now provided, and the total cost of alterations and furnishing is estimated at £9,000. A new mortuary has been erected at the rear of the buildings.

In America the State of Georgia has become noticeable for producing cheap medical schools and cheap doctors. There are five medical schools in the State, and seven others have died after lives of varying length. A Bill was brought forward to regulate the practice of medicine in Georgia, which would doubtless have caused great havoc in the ranks of the profession, and therefore, sufficient pressure being brought to bear, the Bill failed to become law, and Georgian medical men can flourish unrestricted and to the advantage of their patients it is to be hoped.

A fever hospital has just been opened at Stockton. It stands in premises of seven acres. The hospital is designed on the pavilion system. The total cost was £8,000. The Mayor of Stockton at the opening ceremony very wisely reminded those present that expenditure of the rates in respect to a hospital was an expedient and necessary outlay. Previous to the erection of the Stockton Hospital fever patients from that town were treated at Middlesborough, and more recently in a small temporary hospital; both these expedients proving unsatisfactory, Stockton is to be congratulated on having an efficiently equipped and commodious institution of its own.

Some most striking facts in support of revaccination have recently been brought out. Amongst these the statistics as to attendants in small-pox hospitals are particularly valuable. The committee appointed by the Epidemiological Society report that out of 1,500 nurse attendants 43 contracted small-pox, none of whom had been revaccinated. During a past epidemic the only two nurses at the Homerton Hospital who were not revaccinated contracted the disease, and the same experience holds good in respect to other places. At the last meeting of the Leicester Town Council the statistics brought out were all valuable evidence in support of both vaccination and revaccination.

The success of the Liverpool overhead railway will fill many a soiled and heated traveller on the Metropolitan underground railway with feelings of envy and discontent. This electric railroad was opened in March last, and it was regarded by many as a doubtful experiment. The railway company themselves were apparently not without fears, for they sheltered themselves by contracting with the electrical constructors to undertake the working of the line for two years. All doubt, however, is removed now, and electricity has gained one more successful foothold. There can be no question but that if electricity instead of steam could be applied to our underground system throughout, both the health and the comfort of dwellers in our metropolis would be considerably benefited.

